# ﻿A new cherry species (*Prunus*, Rosaceae) from south-western Ecuador

**DOI:** 10.3897/phytokeys.255.151041

**Published:** 2025-04-04

**Authors:** Álvaro J. Pérez, Jorge Andrés Pérez-Zabala, Katya Romoleroux, David A. Espinel-Ortiz, Chaquira Romoleroux, Natasha Albán-Vallejo

**Affiliations:** 1 Herbario QCA, Laboratorio de Botánica Sistemática, Facultad de Ciencias Exactas, Naturales y Ambientales, Pontificia Universidad Católica del Ecuador, Av. 12 de Octubre 1076 y Vicente Ramón Roca, 170525, Quito, Ecuador Pontificia Universidad Católica del Ecuador Quito Ecuador; 2 Herbario Gabriel Gutiérrez Villegas (MEDEL) y Semillero de estudios taxonómicos de plantas de Colombia, Facultad de Ciencias, Universidad Nacional de Colombia, Cra 65, 59A-110, Medellín, Colombia Universidad Nacional de Colombia Medellín Colombia; 3 Bonn Institute of Organismic Biodiversity, University of Bonn, Meckenheimer Allee 170, 53115 Bonn, Germany University of Bonn Bonn Germany

**Keywords:** Buenaventura Reserve, El Oro, Neotropics, plant taxonomy

## Abstract

*Prunusluxurians*, a new species from Buenaventura Reserve at El Oro province in Ecuador is described and illustrated. Additionally, notes on its geographical distribution, ecology, conservation status, and taxonomic affinities are documented. *Prunusluxurians* has some vegetative and floral similarities with other Andean species, but the unique combination of oblong-lanceolate leaves with prominent secondary and tertiary veins, densiflorous floriferous shoots less than 5 cm long, sepals with two marginal glands and flowers with turbinate hypanthium clearly differentiates it from the rest. This is the first species of *Prunus* described from the western flank at elevation below 1500 m, and particularly from a humid spot surrounded by dry areas (Tumbesian influence). Other taxonomic novelties of *Prunus* on this flank can be expected, so further botanical exploration is needed to better understand the diversity of the genus in the region.

## Abstract (es)

Se describe e ilustra *Prunusluxurians*, una nueva especie de la Reserva Buenaventura en la provincia de El Oro en Ecuador. Además, se documentan notas sobre su distribución geográfica, ecología, estado de conservación y afinidades taxonómicas. *Prunusluxurians* comparte algunas similitudes vegetativas y florales con otras especies andinas, pero la combinación única de hojas oblongo-lanceoladas con venas secundarias y terciarias prominentes, brotes floríferos densifloros de menos de 5 cm de largo, sépalos con dos glándulas marginales y flores con hipanto turbinado la diferencian fácilmente del resto. Esta es la primera especie de *Prunus* descrita del flanco occidental a una altitud inferior a 1500 m, y particularmente de un lugar húmedo rodeado de zonas secas (influencia tumbesina). Se espera que existan otras novedades taxonómicas de *Prunus* en este flanco, por lo que es necesaria una mayor exploración botánica para comprender mejor la diversidad del género en la región.

## ﻿Introduction

*Prunus* L. is almost a worldwide distributed genus with around 450 species (Pérez-Zabala 2015) which includes several appreciated fruit crops (e.g. almonds, cherries, plums), ornamentals (e.g. cherry blossom), timber sources (e.g. black cherry) and medicinal ones (e.g. African cherry). It is characterized by a base haploid chromosome number = 8, presence of cyanogenic glycosides, foliar glands, deciduous stipules, monocarpellate solitary pistil and drupaceous fruit (Kalkman 1966; Potter et al. 2007). The species level taxonomy of *Prunus* has been characterized by vague descriptions, minimal or absent taxonomic discussions and typification uncertainty in several old names (McVaugh 1951; Shaw and Small 2004). A major taxonomic revision is particularly complicated considering that around 1230 species’ scientific names have been published under *Prunus*, and more than 2000 names when considering those published under other previously segregated genera and putative hybrids (IPNI 2024). The complexity of the species circumscriptions could be related to natural and human-driven hybridization in some groups (Shaw and Small 2004; Dickinson et al. 2007; Rohrer 2015), underscored phenotypic plasticity (Bortiri et al. 2006), but also to a lack of taxonomic assessment evidenced in many traditional descriptions with limited morphological evaluation that understates potential important traits such as, for instance, leaf venation architecture (Pérez-Zabala 2015) and floral micromorphology (Kalkman 1966).

Although *Prunus* has been traditionally considered as a mainly north temperate genus of deciduous species with marginal diversity of evergreen taxa in mountain regions of the subtropics and tropics (Pennington and Dick 2004), recent studies have shown that similar or even a higher number of species can be found in the tropics (mostly in the Americas, followed by Asia and one species in Africa) from lowlands to around 4000 m (Kalkman 1966; Pérez-Zabala 2015, 2022). All the tropical species and many subtropical ones have been traditionally grouped in the subgenus Laurocerasus (Duh.) Rehd. based on the evergreen condition and the racemose inflorescence. Particularly in the Neotropics, the most important contribution to the knowledge of the genus has been the revisionary work by (Koehne 1915) who recognized 59 taxa, 39 of them as novelties. However, Koehne himself recognized his work as provisional since the revision was based on just 180 specimens mainly stored in European herbaria (most of the species were known from one or few specimens) and made practical sub-treatments with keys by geographic units (countries or group of them), annotating that a consolidated series classification will require further work. After Koehne`s revision, the taxonomic contributions have been mostly isolated publications of new species with no, or minimal, taxonomic discussion, for instance those for Peru (Macbride 1934), Guatemala (Johnston 1938), Colombia (Cuatrecasas 1950), Mexico (Lundell 1968), Paraguay (Basualdo and Zardini 1992) and Venezuela (Li and Aymard 1997). After those works, the last three published novelties of the genus in the region were proposed by Pérez-Zabala (2007) for Colombia based on a previous full taxonomic revision for the country (Pérez-Zabala 2005).

A renewed effort for understanding the diversity and evolution of *Prunus* in the Neotropics has been done recently based on field work and a comprehensive study of more than 4000 collections in herbaria worldwide most of which have been collected during the last 40 years (Pérez-Zabala 2022) and as a result numerous allegedly new species have been identified. Particularly, for the flora of Ecuador, Romoleroux (1996) identified only five native species of *Prunus*, a number that could be considered an underestimation since neighboring countries like Peru and Colombia have more than 30 species each. They also contain the regions where most of the diversity would be expected, along the gradient of the Amazonian flank of the Andes and in inter Andean valleys (Pérez-Zabala 2022). After field work and the examination of some collections from a premontane cloud forest in El Oro province placed in the western flank of the Andes in southern Ecuador, the authors found substantial differences compared to previously published taxa known to the country and with respect to any other species of the genus. Consequently, a new species is proposed here, a full description is provided, morphological differences with similar species are discussed, some ecological aspects of the taxa are examined, and its conservation status is evaluated.

## ﻿Materials and methods

Upon botanical exploration of one of the authors (Á.J.Pérez) in the cloud forest remnants at the Sambotambo Birón area, Reserve Buenaventura, owned and managed by the Jocotoco Foundation (https://www.jocotoco.org.ec) in El Oro province, during December 2021, a small population of an unknown species of *Prunus* was found along the forest remnants borders of the region. To asses taxonomic affinities of the new species we consulted taxonomic literature of South American species and particularly the synopsis of the genus for the Neotropics (Pérez-Zabala 2022), examined specimens at Herbaria GUAY, LOJA, QCA and QCNE (acronyms follow Thiers (2024)), consulted photographic records on iNaturalist (https://www.inaturalist.org), and studied high-resolution images of type material for Neotropical taxa (Tropicos database, https://www.tropicos.org/ and the JSTOR global Plants website http://plants.jstor.org).

The botanical description follows terminology used by Stearn (2004), the leaf morphology type was based on the terms of Hickey and King (2000) and Wilhelm and Rericha (2020), leaf architecture (including venation) was based on Ellis et al. (2009) and glandular position follows Pérez-Zabala (2005). We retained the use of the concept of specialized shoot named flowering shoot instead of the more general term inflorescence, considering the comparable vegetative vs. flower shoot structure in *Prunus* (including both deciduous and evergreen species) in terms of the presence of basal cataphylls homologous to leaves (including fused stipules) (Kalkman 1966), proleptic growth from resting lateral buds developing asynchronously with respect to the subtending leaves (Costes et al. 2014), a predetermined length and number of flowers, and flowers subtended by bracts homologous to reduced leaves (or sometimes well-developed leaves) (Kalkman 1966). Finally, to determine the conservation status of this new species, we followed the guidelines for the use of categories and criteria of the IUCN Red List version 16 (IUCN Standards and Petitions Committee 2024) based on the indicators of criterion B; Geographical distribution is represented as extent of occurrence (B1) and/or area of occupancy (B2).

## ﻿Taxonomy

### ﻿ Prunus luxurians

Taxon classificationPlantaeRosalesRosaceae

Pérez-Zab., Á.J.Pérez, Romol. & N.Albán sp. nov.

899C12C6-0CCF-5353-90F8-EE9DEDA666FF

urn:lsid:ipni.org:names:77359711-1

Figs 1 , 2 , 3

#### Type.

**Ecuador. El Oro**: • Cantón Santa Rosa, Parroquia Torata, Buenaventura Reserve, Jocotoco Foundation, 03°33'54.7"S, 79°46'47.5"W, 1300–1400 m, 22 Dec 2021 (fl), *Á.J. Pérez, P. Mena-Olmedo, A. de la Cruz, J. Zambrano & L. Aguilar 11743* (holotype: QCA; isotypes: LOJA, QCNE).

#### Diagnosis.

*Prunusluxurians* has a unique combination of leaves oblong lanceolate, cernuous in posture, around three times longer than wider, with prominent secondary and tertiary veins, secondary veins curved toward the margin and apparently eucamptodromous but becoming brochidodromous distally, base rounded to subcordate, floriferous shoots erect, densiflorous, less than 5 cm long, sepals with 2 marginal glands and flowers with turbinate hypanthium. *Prunusintegrifolia* (Presl.) Walp. share with the new species the leaf shape and transverse posture, number of secondary veins, erect floriferous shoots and anthers of similar size; but, the new species has leaves shorter than 14 cm (vs. more than 15 cm.), chartaceous (vs. coriaceous), with conspicuous venation (vs. relatively obscure), glands submarginal and separated from the midrib (vs. attached to the midrib), turbinate hypanthium (vs. wide turbinate) and erect pedicels (vs. recurved) (Fig. 4).

**Figure 1.**
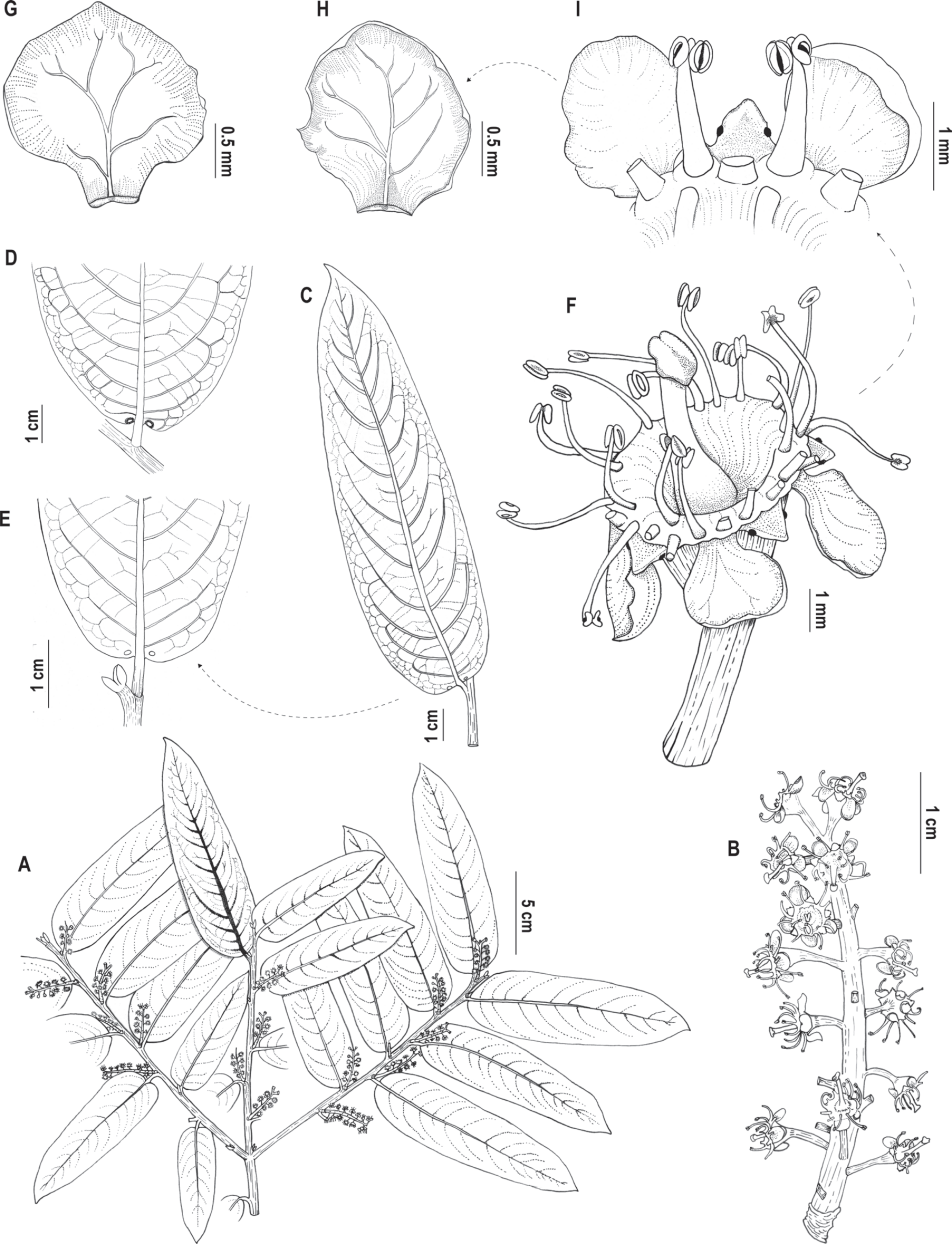
*Prunusluxurians* Pérez-Zab., Á.J.Pérez, Romol. & N.Albán **A** habit **B** floriferous shoot **C** leaf lower surface **D, E** leaf glands **F** flower **G** petal lower surface **H** petal upper surface **I** detail of petals and sepals (notice marginal glands). **A–I** based on *Á.J. Pérez et al. 11743* (QCA). Illustrations by Natasha Albán.

**Figure 2.**
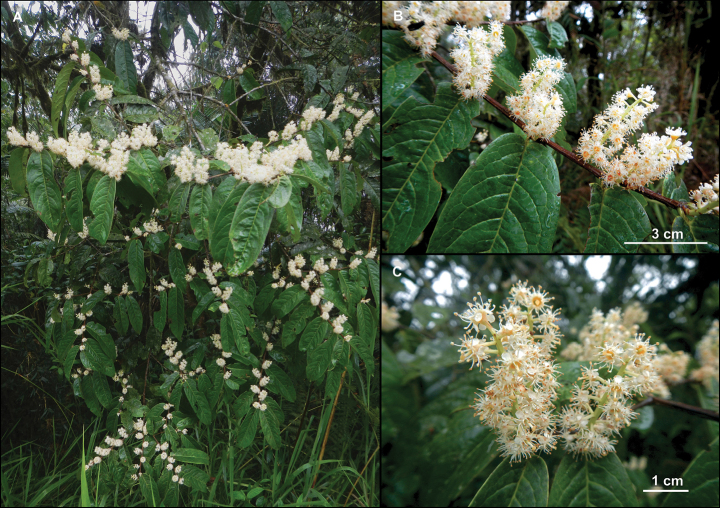
*Prunusluxurians* Pérez-Zab., Á.J.Pérez, Romol. & N.Albán **A** habit **B** branch with leaves and floriferous shoots **C** flowers. Photos by Á.J. Pérez.

**Figure 3.**
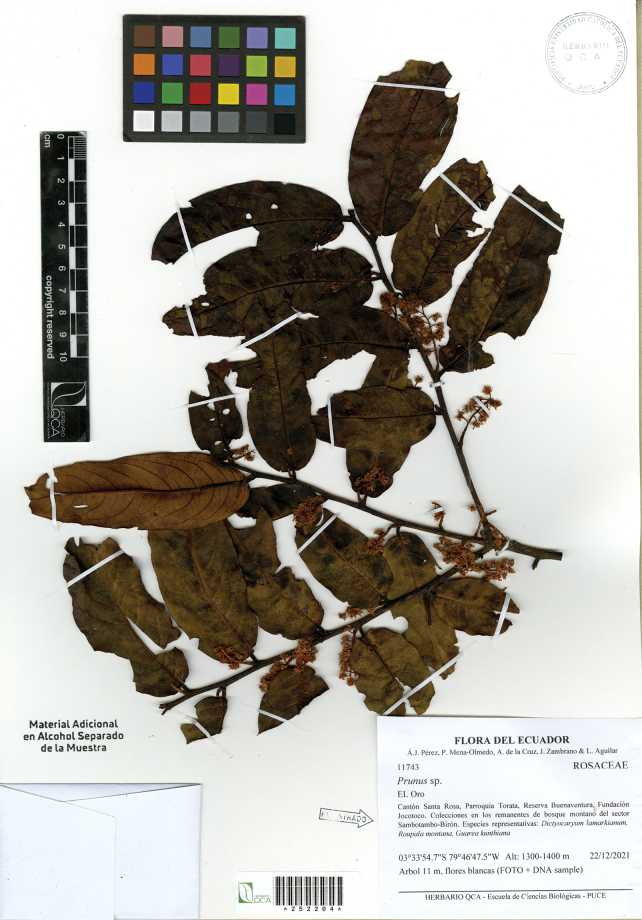
*Prunusluxurians* Pérez-Zab., Á.J.Pérez, Romol. & N.Albán holotype collection *Á.J. Pérez et al. 11743* (QCA).

**Figure 4.**
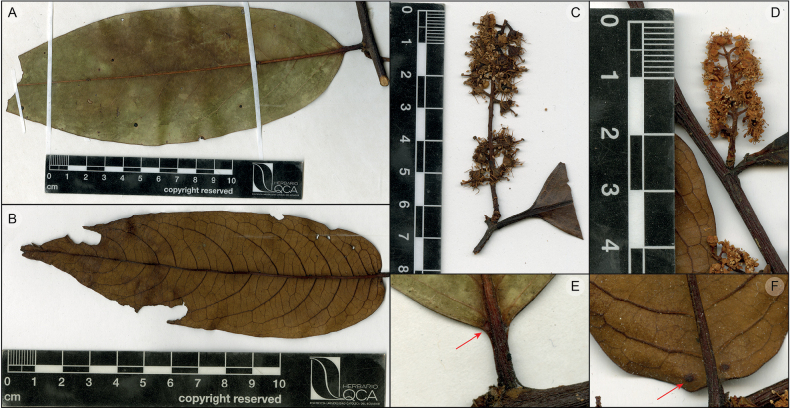
Differences between *Prunusluxurians* Pérez-Zab., Á.J.Pérez, Romol. & N.Albán (**B**, **D**, **F**) and *Prunusintegrifolia* (Presl.) Walp. (**A**, **C**, **E**). **A, B** leaf abaxial surface **C, D** floriferous shoots **E, F** leaf gland. **A**, **E** sample *Lægaard 17716* (QCA), **B**, **D**, **F***Á.J. Pérez et al. 11743* (QCA), and **C***Salinas 3188* (QCA).

#### Description.

***Tree*** up to 11 m tall; main trunk bark brown, grooved, with brown to light brown lenticels. Most recent growth units 2–2.6 mm diam. at base, with 8–9 leaves per unit, glabrous, angled, reddish brown in vivo; lenticels (0.2–) 0.5–0.9 × 0.1–0.4 mm, elliptic to narrowly elliptic, slightly protuberant, density up to 32 per centimeter (at the base of last growth units); cataphylls 2.8–3 × 3.5–4.1 mm, trapezoidal, trilobed at the apex, glabrous. ***Leaves*** alternate, distichous on plagiotropic shoots, transversal posture cernuous when fresh; petioles 3.5–5.9 × 1–1.4 (–1.8) mm long, flexuous, longitudinally grooved, glabrous; stipules paired, 1.3–1.5 mm at the base (scar), ca. 3.5 mm long, oblong falcate, apex acute, apparently deciduous when blades start expanding; leaf-blades (6.1–) 10–13.65 × (2.9–) 3.5–4.65 cm, around three times longer than wide, oblong-lanceolate, equilateral, chartaceous, conduplicate longitudinally, base rounded to subcordate, apex acute; margin entire to slightly repand; upper surface lustrous, impressed to slightly bulliform, glabrous; lower surface opaque, slightly rugose, glabrous; midrib 0.6–0.9 mm wide, slightly depressed above, prominent below, secondary vein framework apparently eucamptodromous but becoming brochidodromous distally, secondary veins 7–14 pairs, departing at 70–80 degrees from the midrib and progressively curving up to 30 degrees after the first quarter and almost parallel to the margin towards the end, slightly depressed above, raised below; intersecondaries 1–3 per intercostal area, following up the proximal trajectory of secondaries up to 20–30% of their length; tertiary intercostal opposite percurrent sinuous, obtuse with respect to the midvein, epimedial tertiary alternate percurrent and perpendicular to midvein, distal course basiflexed, slightly depressed above, impressed below, exterior tertiary veins looped; quaternary and higher order veins inconspicuous; leaf glands 2, elliptic to circular, 0.5–1 × 0.5–1 mm, dark brown, sub-basal below the first pair of secondary veins, distanced (0.3–) 1–1.2 (–3.4) mm from the midrib and 0.2–0.9 mm from the margin. ***Floriferous shoots*** with a single axis, erect to suberect, present on the axils of all leaves and cataphylls of the current growth units, axis (17–) 32–44.5 × 2.4–2.8 mm at the base and keeping a similar width until the middle, light green in vivo, glabrous, 14–27 flowered with groups of 3–4 flowers closer together; first flower at ca 8 mm from the base; cataphylls 1.6–3.7 × 1.2–4.1 mm, glabrous, trapezoidal, floral bracts not seen. ***Flowers*** pedicellate, pedicels 1.5–5.4 × 0.3–1.4 mm, straight, thickened at apex, white, glabrous; hypanthium 1.5–2.1 × 2–3.5 mm, turbinate, pale yellow, glabrous outside; sepals 0.7–1.2 × 0.7–1.5 mm, broadly triangular, margin entire, apex acute, glabrous outside and inside, slightly reflexed, with two marginal subapical glands that turn black when dry; petals 1.5–2.4 × 1.1–2 mm, widely obovate, 0.4–0.6 (–0.8) mm wide at the basal claw, white, glabrous, apex rounded, margin entire, and slightly involute, venation with 4–6 lateral branches little conspicuous, eglandular; stamens 19–26, in two series, the outer series (3–3.2 mm long) longer than the inner one (1.5–2 mm long), glabrous, filaments 1.4–3.5 mm long, white, anthers 0.4–0.6 × 0.3–0.4 mm, basifixed with indorse dehiscence, elliptic-oblong, yellow; pistil 2–4.5 mm long, ovary 0.3–1.2 × 0.2–1.4 mm, glabrous, style 0.3–2.7 mm long, as long as or shorter than stamens, glabrous, stigma 0.5–1.0 mm wide, discoid-lobed. ***Fruit*** not seen.

#### Etymology.

We use the epithet *luxurians* to refer to the profuse blooming and outstanding beauty of this species at its flowering time that make it very conspicuous at the lower and mid-strata of the forest where it inhabits.

#### Distribution, habitat and ecology.

**Figure 5.**
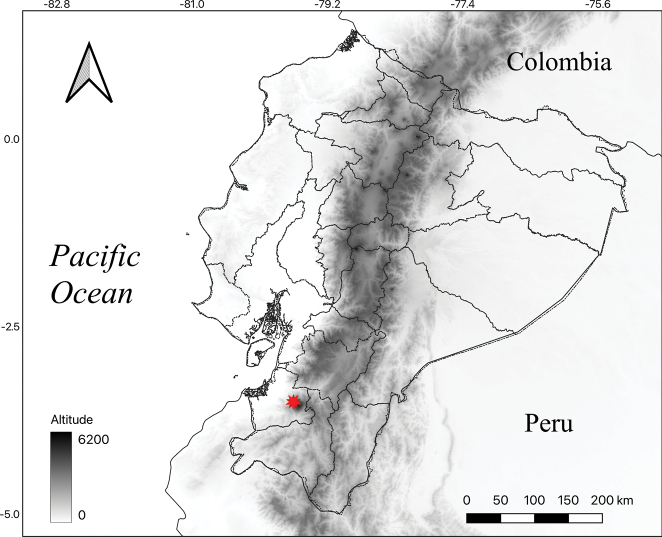
Distribution map of *Prunusluxurians* Pérez-Zab., Á.J.Pérez, Romol. & N.Albán (red star) from south-western Ecuador. Map generated by Á.J. Pérez & E. Rea.

Known thus far only from the type locality in El Oro province, south-western Ecuador, a montane forest remnant at the Sambotambo-Birón area along the road to Cerro Pelado, between 1300–1600 m (Fig. 5). According to the Ministerio del Ambiente del Ecuador (2013) the type locality lies in the Catamayo-Alamor evergreen piedmont forest (BsPn02) that harbors high diversity and endemism (Cerón et al. 1999; Myers et al. 2000) as a result of the Andes and Tumbesian region influence. In the Sambotambo-Birón area a total of five individuals of *Prunusluxurians* were observed growing on the borders of the forest remnants co-occurring with the following tree species: *Guatteriamicrocarpa* Ruiz & Pav. (Annonaceae), *Dictyocaryumlamarckianum* (Mart.) H.Wendl., *Wettiniakalbreyeri* (R.Bernal) R.Bernal (Arecaceae), *Guareakunthiana* A.Juss. (Meliaceae), *Roupalamontana* Aubl. (Proteaceae) and the recently described *Magnoliabuenaventurensis* Á.J.Pérez & E.Rea (Magnoliaceae) and *Begoniajocotocoi* Á.J.Pérez & Tebbitt (Begoniaceae) (Pérez et al. 2023, 2024).

#### Phenology.

Flowering from October to January and probably fruiting in July to August (the latter according to testimonies of people living in the area).

#### Conservation status.

Known only from a single herbarium collection of a population of around five adults that we observed growing on the edges of the forest remnants in the Buenaventura Reserve, close to the road to Cerro Pelado in the Sambotambo-Birón area. Based on the available information, and according to the IUCN Red List Criteria and Guidelines (IUCN Standards and Petitions Committee 2024), *Prunusluxurians* is preliminarily assessed as Critically Endangered (CR B2ab(iii)) because of its limited area of occupancy (AOO < 5 km2) and a single known population of around five individuals. Additionally, mining and farming activities currently threaten the forest remnants around the type locality. Priority reproduction studies and germination assays are needed for attempting *ex-situ* conservation of this promising ornamental species.

## ﻿Discussion

*Prunusluxurians* has similarities with some Andean species that display oblong lanceolate leaves, transverse posture cernuous, secondary veins exmedially curved and apparently eucamptodromous but distally becoming brochidodromous, two leaf glands placed around the leaf base, floriferous shoots erect or suberect, and anthers around 0.5 mm long like *P.integrifolia*, *P.rugosa* Koehne, *P.pearcei* Rusby and *P.pleiantha* Pilger. However, all these four species have larger leaves (generally starting from 15 cm), glands attached to the midrib, longer flowering shoots (which may be also subflexuous) and recurved floral pedicels. Other apparently eucamptodromous species are *P.schultzeae* Pilger from the Amazon foothills below 500 m in Pastaza and Napo (Ecuador), and *P.sana* Macbr. and *P.williamsii* Macbr. from mid-elevation (700–1200 m) Amazon flanks in northern Peru and Ecuador, and Cuzco in central Peru, respectively. However, they all have elliptic leaves with acuminate to very acuminate apex, impressed secondary veins, and little conspicuous tertiary veins, flexuous floral axis, and smaller flowers with all parts smaller than *P.luxurians*. On the other hand, other Andean species with oblong to lanceolate leaves (*P.opaca* (Benth.) Walp. and *P.littlei* Pérez-Zab.) generally have smaller leaves (up to 10 cm long), a clearly brochidodromous secondary vein framework (relatively impressed and then obscure when dry), flexuous floral axes and a campanulate hypanthium. Finally, this is the first tropical species in which glands have been observed in the sepals, but certainly, this character could have been unnoticed in previous descriptions and may be present in some other Neotropical species. Marginal sepal glands (sometimes as part of tooth) are present in Eurasian species like *P.armeniaca* L., *P.cerasifera* Ehrh., *P.cerasus* L., *P.domestica* L., *P.glandulosa* Thunb., *P.spinosa* L., *P.subhirtella* Miq., *P.yedoensis* Matsum., and in the North American species *P.americana* Marshall, *P.andersonii* A. Gray, *P.fremontii* S. Watson, *P.hortulana* L.H. Bailey, *P.mexicana* S. Watson, *P.murrayana* E.J. Palmer, *P.nigra* Aiton, *P.rivularis* Scheele, *P.subcordata* Benth., and *P.texana* D. Dietr. (Rohrer 2015).

Most of the species with a wide range of occurrences in the Andes grow above 1800 m (ca *P.integrifolia*, *P.littlei* and *P.opaca*), whereas species described from the eastern foothills of the Andes below 1800 m have a relatively restricted distribution (ca *P.amplifolia* Koehne, *P.sana*, *P.williamsii*). *Prunusluxurians* is the first species of *Prunus* to be described from the western flanks, at an altitude of less than 1500 m, and in particular from a humid spot surrounded by dry areas (the latter known as the Tumbesian influence). Only three species of *Prunus* are known from the west flanks of the Andes: *Prunusmegacarpa* Pérez-Zab, *P.rigida* Koehne and *P.subcorymbosa* Koehne. *Prunusmegacarpa* grows in very humid forests between 1600 and 1900 m in northwestern Colombia, and is characterized by obovate leaves, multiple leaf glands and very large fruits. *Prunusrigida* is found in humid or sub-humid forest or shrublands above 2800 m from Cajamarca to Lima, and has entire to dentate leaves, impressed venation, erect floral axis and anthers larger than 1 mm long. The last species, *P.subcorymbosa*, is widely distributed from Bolivia to Venezuela from 800 to 2500 m, and it has pubescent buds, young leaves and floral axes, flowering shoots generally branched from the axils of the cataphylls, hypanthium widely campanulate and pubescent, and fruits prolate and longer than 15 mm. The western flanks of the Andes in Colombia and Ecuador have been poorly explored although a high diversity is expected due to the presence of the hyperdiverse extremely humid Choco biogeographic region from eastern Panama to central Ecuador (Pérez-Escobar et al. 2019), and the special local climatic conditions found in the transition from humid to dry forest in southern Ecuador (Cerón et al. 1999). Few collections of *Prunus* have been recorded from the western flanks of the Andes, and some of those seen by the authors correspond to taxonomic novelties, indicating that further botanical exploration is necessary to get a better picture of the diversity of the genus in the region.

## Supplementary Material

XML Treatment for Prunus luxurians

## ﻿Additional information

### Author ORCIDs

Álvaro J. Pérez https://orcid.org/0000-0002-0644-9111

Jorge Andrés Pérez-Zabala https://orcid.org/0000-0003-1388-7694

Katya Romoleroux https://orcid.org/0000-0002-0679-9218

David A. Espinel-Ortiz https://orcid.org/0000-0003-3405-0375

Chaquira Romoleroux https://orcid.org/0009-0003-0278-3790

Natasha Albán-Vallejo https://orcid.org/0009-0008-1506-8768

### Data availability

All of the data that support the findings of this study are available in the main text.
